# The Role and Mechanism of Erythrocyte Invasion by *Francisella tularensis*

**DOI:** 10.3389/fcimb.2017.00173

**Published:** 2017-05-09

**Authors:** Deanna M. Schmitt, Rebecca Barnes, Taylor Rogerson, Ashley Haught, Leanne K. Mazzella, Matthew Ford, Tricia Gilson, James W.-M. Birch, Anders Sjöstedt, Douglas S. Reed, Jonathan M. Franks, Donna B. Stolz, James Denvir, Jun Fan, Swanthana Rekulapally, Donald A. Primerano, Joseph Horzempa

**Affiliations:** ^1^Department of Natural Sciences and Mathematics, West Liberty UniversityWest Liberty, WV, USA; ^2^Department of Clinical Microbiology, Clinical Bacteriology, and Laboratory for Molecular Infection Medicine Sweden, Umeå UniversityUmeå, Sweden; ^3^Regional Biocontainment Laboratory, Center for Vaccine Research, University of PittsburghPittsburgh, PA, USA; ^4^Center for Biologic Imaging, University of Pittsburgh School of MedicinePittsburgh, PA, USA; ^5^Department of Biomedical Sciences, Joan C. Edwards School of Medicine, Marshall UniversityHuntington, WV, USA

**Keywords:** erythrocyte invasion, tularemia, type VI secretion system, tick borne disease, spectrin

## Abstract

*Francisella tularensis* is an extremely virulent bacterium that can be transmitted naturally by blood sucking arthropods. During mammalian infection, *F. tularensis* infects numerous types of host cells, including erythrocytes. As erythrocytes do not undergo phagocytosis or endocytosis, it remains unknown how *F. tularensis* invades these cells. Furthermore, the consequence of inhabiting the intracellular space of red blood cells (RBCs) has not been determined. Here, we provide evidence indicating that residing within an erythrocyte enhances the ability of *F. tularensis* to colonize ticks following a blood meal. Erythrocyte residence protected *F. tularensis* from a low pH environment similar to that of gut cells of a feeding tick. Mechanistic studies revealed that the *F. tularensis* type VI secretion system (T6SS) was required for erythrocyte invasion as mutation of *mglA* (a transcriptional regulator of T6SS genes), *dotU*, or *iglC* (two genes encoding T6SS machinery) severely diminished bacterial entry into RBCs. Invasion was also inhibited upon treatment of erythrocytes with venom from the Blue-bellied black snake (*Pseudechis guttatus*), which aggregates spectrin in the cytoskeleton, but not inhibitors of actin polymerization and depolymerization. These data suggest that erythrocyte invasion by *F. tularensis* is dependent on spectrin utilization which is likely mediated by effectors delivered through the T6SS. Our results begin to elucidate the mechanism of a unique biological process facilitated by *F. tularensis* to invade erythrocytes, allowing for enhanced colonization of ticks.

## Introduction

*Francisella tularensis* is a highly infectious gram negative bacterium that causes the disease tularemia (Dennis et al., 2001; Nigrovic and Wingerter, 2008). The Center for Disease Control and Prevention classifies *F. tularensis* as a Category A bioterrorism agent due to the low infectious dose (<10 bacteria) and high degree of mortality (approaching 60% in untreated individuals) associated with this bacterium (Dennis et al., 2001). In addition to being a potential agent of bioterror, *F. tularensis* causes a number of naturally occurring zoonoses including oropharyngeal, ocular, and ulceroglandular diseases (Ellis et al., 2002). Most commonly, human tularemia is acquired through contact with infected game animals (Gurcan, 2014). Blood sucking arthropods, such as ticks, are responsible for transmitting the bacteria among the mammalian reservoirs in the wild (Morner, 1992; Gurcan, 2014). However, ticks and other blood sucking arthropods can also spread tularemia to humans—which is especially evident in endemic areas, such as Turkey, Sweden, and parts of the United States (Morner, 1992; Feldman et al., 2001; Sjostedt, 2007).

The pathogenesis of *F. tularensis* has primarily been attributed to the ability of this bacterium to replicate within phagocytic cells of the innate immune system, such as macrophages (Barker and Klose, 2007). However, *F. tularensis* can also invade and replicate in a range of non-phagocytic host cells, such as alveolar epithelial cells, kidney epithelial cells, hepatocytes, and fibroblasts (Fujita et al., 1993; Qin and Mann, 2006; Hall et al., 2007, 2008; Craven et al., 2008; Horzempa et al., 2008). These alternative host cells also appear to be important in sustaining the infection as replication in non-macrophages is sufficient to mediate the pathogenesis of *F. tularensis* (Bosio and Dow, 2005; Horzempa et al., 2010a). We have shown that *F. tularensis* invades erythrocytes (Horzempa et al., 2011), which are host cells incapable of undergoing phagocytosis or endocytosis (Schekman and Singer, 1976). In various other pathogenic protozoa and bacteria, it has been speculated that intraerythrocytic infection facilitates persistence of the pathogen allowing for efficient transmission by arthropods (Carter, 2001; Schulein et al., 2001; Shaw, 2003). As arthropods are also key vectors for transmission of tularemia (Petersen et al., 2009), habitation of red blood cells (RBCs) may facilitate successful colonization of these vectors by *F. tularensis*.

Uptake of *F. tularensis* by both macrophages and non-phagocytes is mediated by the host cell's endocytic machinery (Clemens et al., 2005; Craven et al., 2008). Erythrocytes, however, do not undergo endocytosis (Schekman and Singer, 1976) and possess a unique cytoskeleton consisting of a meshwork of flexible spectrin filaments held together by short f-actin bundles (Palek and Liu, 1980; Chakrabarti et al., 2006; Dhermy et al., 2007). The distinct structural differences between these host cell types suggest *F. tularensis* likely produces specific bacterial factors to manipulate the unique erythrocyte cytoskeleton for entry. In *Bartonella* spp. and *Anaplasma marginale*, initial attachment to RBCs was shown to be critical for subsequent invasion (Kocan et al., 2007; Dehio, 2008). In *Bartonella*, this process is mediated through an adhesion event involving genes that encode for components of the Trw Type VI secretion system (T6SS) (Dehio, 2008). *F. tularensis* does not encode any proteins with significant sequence similarity to Trw or others involved in erythrocyte invasion by *Bartonella* or *Anaplasma* suggesting this bacterium utilizes a unique mechanism of internalization (Horzempa et al., 2011). Both the virulent *F. tularensis* type A strain (Schu S4) and the attenuated type B strain (LVS) invade human erythrocytes *in vitro* at a similar rate (Horzempa et al., 2011). Importantly, this suggests that molecular mechanisms mediating this process are likely shared among these strains.

In this study, we investigated the role of erythrocyte invasion in tularemia pathogenesis and tick transmission, and identified host and bacterial factors responsible for mediating this phenomenon.

## Materials and methods

### Cultivation of bacteria

Bacterial strains used in this study are listed in Table 1. Frozen stock cultures of bacteria were streaked onto chocolate II agar plates and incubated at 37°C with 5% CO_2_ for 1–3 days. These bacteria were used to inoculate either Chamberlain's chemically defined medium (CDM; Chamberlain, 1965), tryptic soy broth (Becton Dickinson and Co.) supplemented with 0.1% cysteine hydrochloride (Fisher Scientific) (TSB-C), or brain heart infusion broth (BHI; Oxoid Ltd.) (adjusted to pH 6.8; Hazlett et al., 2008) and grown to stationary phase by an overnight incubation at 37°C with agitation. All work with the Schu S4 strain was conducted under BSL-3 conditions at the University of Pittsburgh with approval from the CDC Select Agent Program.

**Table 1:** **Bacterial strains, plasmids, and primers used in this study**.

**Strain, plasmid, or primer**	**Description**	**Source or reference**
***F. tularensis*** **STRAINS**		
LVS	*F. tularensis* subsp. *holarctica* live vaccine strain	Karen Elkins
Schu S4	*F. tularensis* subsp. *tularensis* Schu S4	BEI Resources
Δ*mglA*	LVS with a deletion of *mglA*	
*iglC* null	LVS with both copies of *iglC* deleted	This study
*iglC* null::pTG28	LVS *iglC* null trans-complement	This study
Δ*dotU*	LVS with a deletion of *dotU*	
Δ*dotU*::pDotU	LVS Δ*dotU* trans-complement	
***E. coli*** **STRAINS**		
DH5α	*fhuA2 Δ(argF-lacZ)U169 phoA glnV44 Φ80 Δ(lacZ)M15 gyrA96 recA1 relA1 endA1 thi-1 hsdR17*	NEB
**PLASMIDS**		
pJH1	pMQ225 with the I-SceI restriction site	
pTG1	pJH1 with 1-kb DNA from both the up- and downstream adjacent regions flanking the *iglC* chromosomal locus	This study
pGRP	pFNLTP5 with the FTL_0580 (FGRp) promoter	
pGUTS	pGRP with I-SceI under the control of FGRp	
pTG28	pGRP containing *iglC* under the control of FGRp	This study
**PRIMERS**		
iglC1	5′- CATGGCATGCTAAGATTGGTAGTATTGTGGATGTCGAGTCG-3′	IDT
iglC2	5′-GTCGACGGTACCACCGGTTTATTATTAACTAGCAGCAGCTGTAGCCG-3′	IDT
iglC3	5′-TAATAATAAACCGGTGGTACCGTCGACCTATCTAATTTAGAGTTATATCCAATAAGTGC-3′	IDT
iglC4	5′-CATGCTGCAGCTTATCAGTCATTATTTGTAAAGATAACGG-3′	IDT
conf 1F	5′-CGATCGTAGGGATAACAGGGTAATG-3′	IDT
conf 1R	5′-CAATTACTTTCTCTCTGATATTCAGAAATAAG-3′	IDT
conf 2F	5′-CCCGGGGATCCTCTAGAGTCG-3′	IDT
conf 2R	5′-GTGTATATCGAGAATATCTCATTATATTAAATGTG-3′	IDT

*IDT, Integrated DNA Technologies*.

### Mice

Six- to eight-week old female C57BL/6J mice purchased from Jackson Laboratories (Bar Harbor, ME) were housed under ABSL-3 conditions in the University of Pittsburgh Regional Biocontainment Laboratory (RBL). All research involving animals was approved by the University of Pittsburgh's Institutional Animal Care and Use Committee.

### Erythrocyte isolation

Murine erythrocytes were isolated from blood obtained in a heparinized syringe via cardiac puncture (Horzempa et al., 2011). Human RBCs were isolated from buffy coats obtained from Pittsburgh Central Blood Bank, with approval from the institutional review board (Horzempa et al., 2011). The buffy coat or blood sample was then mixed with an equal volume of phosphate-buffered saline (PBS; Cellgro) and separated by density gradient centrifugation using Ficoll (GE Heatlthcare Bio-sciences) (Noble and Cutts, 1967). As an alternative, Lympholyte-Poly (Cedarlane) was used to separate erythrocytes from granulocytes, mononuclear cells, and plasma (Boyum, 1964, 1968; Ferrante and Thong, 1980). Erythrocytes were then washed and suspended in McCoy's 5A (M5A) medium (Cellgro) supplemented with 10% human AB serum (Gemini Bio-Products) and 25 mM HEPES (Cellgro) (complete M5A).

### Gentamicin protection assay

Erythrocytes were suspended in complete M5A and aliquoted into a 96-well V-bottom microtiter dish (Nunc). In select experiments, cells were treated with the indicated molecular agents [cytochalasin D (MP Biomedicals), phalloidin (EMD Millipore Calbiochem), *Pseudechis guttatus* snake venom (Sigma Aldrich), or mitoxantrone (Fisher Scientific)] for 20–30 min and washed with PBS prior to incubation with *F. tularensis*. Bacteria from overnight broth cultures were suspended in complete M5A and incubated at 37°C for 20 min prior to infection. Bacteria were then added to the erythrocytes and incubated together at 37°C with 5% CO_2_ for 2–3 h. The initial bacterial density used for these infections was estimated based on OD_600_ of bacteria grown to stationary phase where we had determined that OD_600_ of 0.3 is equivalent to 5 × 10^8^ CFU/ml. The actual multiplicity of infection (MOI) was determined by diluting and plating for CFU. Data generated from experiments in which the actual MOI fell within the range of 5–100 were used in the current study. To kill the remaining extracellular bacteria, RBCs were pelleted (100 × g, 5 min) and treated with gentamicin (US Biological; 100 μg/mL in PBS) for 1 h at 37°C with 5% CO_2_. In select experiments, erythrocytes were instead suspended in pH-adjusted complete M5A or treated with proteases associated with digestive cells of the tick gut (Sojka et al., 2013) [legumain (R&D Systems; 0.55 mg/ml), papain (1.2 mg/ml), and cathepsin D (Invitrogen; 0.1 mg/ml)] as indicated. Cells were washed with complete M5A and then lysed using 0.02% SDS (Hoefer, Inc.). This suspension was diluted and plated to enumerate colony forming units (CFU). Controls included wells containing bacteria but not RBCs (Horzempa et al., 2011). Stock solutions of cytochalasin D (Cyt) (100 μg/mL in chloroform), phalloidin (16 μM in ethanol), mitoxantrone (Mtx) (100 μg/mL in Deionized H_2_O) were stored at −20°C and diluted to the desired concentration in complete M5A the day of the experiment. Freeze-dried *P. guttatus* venom was reconstituted at 25 μg/mL in complete M5A on the day utilized.

### Intravenous mouse infections

*Francisella tularensis* Schu S4-infected murine erythrocytes recovered from a gentamicin protection assay were washed, suspended in PBS, and administered to naïve mice by tail vein injection (~1.5 × 10^6^ erythrocytes/mouse). As controls, mice were infected intravenously with either Schu S4 liberated from erythrocytes or mock-infected erythrocytes mixed with Schu S4 immediately before infection. Mice were monitored twice daily for morbidity and mortality. Morbidity was measured by a nominal scale that integrated grooming, activity, posture, and overall appearance of the mice. Once a predetermined score was achieved, the mice were euthanized.

### *In vitro* tick feeding and bacterial colonization

Adult *Amblyomma americanum* and *Ixodes scapularis* ticks were purchased from Oklahoma State University tick rearing facility. These ticks were selected because they have been shown to feed robustly *in vitro*. Moreover, *A. americanum* ticks transmit tularemia in the US and related species of *Ixodes* ticks have been shown to harbor *F. tularensis* in Europe and parts of Asia (Rechav et al., 1999; Castellaw et al., 2010; Brown et al., 2011; Dobler et al., 2014). An *in vitro* method using glass capillary tubes (Rechav et al., 1999) was used to feed ticks human erythrocytes containing *F. tularensis* LVS or bacteria that had been liberated from these host cells. Briefly, the dorsal side of the tick was attached to a glass slide using double-sided tape. Erythrocytes were prepared and were incubated with *F. tularensis* LVS as previously described for 2–3 h to allow for invasion to occur. RBCs were either washed complete M5A or washed and lysed as previously described to liberate intracellular bacteria and then were fed to ticks for 2 h while being incubated at 37°C, 5% CO_2_. The lysis step did not reduce the number of viable bacteria being fed to ticks (data not shown). At the time points indicated, ticks were homogenized in 850 μl 0.02% SDS using a sterile mortar and pestle, and homogenates were plated on a medium selective for *Francisella* [Chocolate II agar containing antibiotics Vancomycin (12.5 μg/ml), Ampicillin (100 μg/ml), and Polymixin-B (100 μg/ml)]. After incubation for 3 days at 37°C with 5% CO_2_, colonies were counted to determine CFU/tick. At least 3 ticks were fed per group and all experiments were repeated 2–3 times.

### Transmission electron microscopy (TEM) with immunogold labeling

Human erythrocytes were co-cultured with *F. tularensis* Schu S4 for 4 h and then subjected to a gentamicin protection assay as described above. The longer incubation time was to increase the chances of observing *Francisella*–erythrocyte interactions. Subsequently, cells were washed, fixed in 2% paraformaldehyde plus 0.1% glutaraldehyde in PBS, and then prepped for TEM as described previously (Horzempa et al., 2011). Sections were labeled with primary (polyclonal rabbit anti-*F. tularensis;* BD Biosciences) and gold conjugated secondary antibodies (goat anti-rabbit 5 nm; Amersham). After labeling, sections were washed in PBS containing 0.5% bovine serum albumin and 0.15% glycine and PBS alone, and then fixed in 2.5% glutaraldehyde in PBS. Sections were post-stained in 2% neutral uranyl acetate for 7 min, washed three times in ddH_2_O, stained for 2 min in 4% uranyl acetate, and embedded in 1.25% methyl cellulose. Sections were viewed under a JEOL JEM 1011 electron microscope at 80 kV fitted with a side mount AMT 2k digital camera.

### RNA extraction and RNA-Seq library preparation

To identify bacterial genes differentially expressed in the presence of erythrocytes, RNA was extracted from *F. tularensis* LVS that was incubated in the presence or absence of human erythrocytes (five replicate cultures per group) in a similar fashion as we described above. To collect RNA exclusively from bacterial cells, erythrocytes were lysed with deionized water while the intact bacterial cells (exposed and control cells) were then collected by centrifugation. Total RNA was extracted using a Qiagen RNeasy kit; RNA integrity (RIN-value) was assessed by electrophoretic analysis under an Agilent Bioanalyzer protocol. RIN-values were 8 or greater for all samples. LVS RNA-Seq libraries were prepared from 1,000 ng total RNA using an Epicentre ScriptSeq Complete Kit which is designed to deplete ribosomal RNA from gram-negative organisms and allow for construction of barcoded, Illumina-compatible RNA-Seq libraries. Libraries were sequenced in a 2 × 50 base paired end strategy on an Illumina HiSeq1500 sequencer. Total reads per library ranged from 25,878,644 to 32,410,910. Mean Illumina Q scores ranged from 36.48 to 37.9.

### RNA-Seq analysis

Demultiplexing of samples was performed using CASAVA 1.8.2 (Illumina). Reads were aligned to the *F. tularensis* subsp. *holarctica* LVS (NCBI accession: NC_007880.1) genome using Bowtie2-2.2.5 (Langmead et al., 2009) and SAMtools-1.2 (Li et al., 2009). Genes differentially expressed between control and erythrocyte-exposed samples were identified using the Bioconductor/R package DESeq2 (Love et al., 2014), with an adjusted *p*-value (false discovery rate) of 10% or less being considered differentially expressed. Raw RNA-Seq data were submitted to the NCBI Gene Expression Omnibus (GEO) and are accessible via accession number GSE93233.

### Construction of *F. tularensis* LVS *iglC*-null mutant and complement

All primers and plasmids used in this study are listed in Table 1. The *F. tularensis* LVS *iglC*-null mutant and complement were generated using methods described previously (Horzempa et al., 2010a,b). To preserve operon integrity and any potential regulatory sites within the target gene sequence, the entire *iglC* gene was not deleted. Rather, codons 71–163 (of 209) were deleted and three consecutive stop codons (ochre) were inserted after codon 70. To begin, the *iglC* deletion construct pTG1 was generated using splicing by overlap-extension PCR. Regions (~500 bp) upstream and downstream of the *iglC* sequence targeted for deletion were amplified by PCR using the primer pairs iglC1 with iglC2 and iglC3 with iglC4, respectively. The resulting amplicons contained regions of overlap and served as template DNA for a second PCR reaction using primers iglC1 and iglC4. The resulting 1,000 bp fragment was then TA-cloned into pGEM (pGEMΔi*glC*). This construct was digested with SphI/PstI, and the fragment containing the *F. tularensis* DNA was ligated into pJH1, which had been digested with the same enzymes, to produce pTG1. This vector was then transferred into *F. tularensis* LVS by tri-parental mating as described previously (Horzempa et al., 2010b). Merodiploid strains were recovered and transformed with pGUTS by electroporation to allow for expression of I-SceI which causes a double-stranded break forcing recombination and allelic replacement (Horzempa et al., 2010a,b). Colonies resistant to kanamycin were screened by PCR for deletion of one copy of the *iglC* gene using primers iglC1 and iglC4. Colonies with a single *iglC* deletion produced two amplicons, one including the full-length *iglC* gene and another reduced in size by ~250 bp. To cure pGUTS, the *F. tularensis* Δ*iglC* strains were passaged at least twice in TSBc, diluted, and plated to a density of 100 to 300 CFU per chocolate II agar plate. Plates were incubated for at least 3 days at 37°C, 5% CO_2_ and colonies that formed were replica plated onto chocolate II agar plates with and without kanamycin. Those colonies sensitive to kanamycin were isolated and again tested for sensitivity to this antibiotic. The resulting LVS strain contained one full-length copy of *iglC* and one truncated copy missing the central 93 codons of *iglC* (LVS/Δ*iglC*1).

Because *F. tularensis* naturally contains two copies of the targeted gene, the second copy of *iglC* in LVS/Δ*iglC*1 was deleted using similar methods as described above. Merodiploids were screened by PCR (primers conf 1F with conf 1R and conf 2F with conf 2R; conf 1F; and conf 2F anneal with the vector portion of pTG1 while conf 1R and conf 2R anneal to the *Francisella* chromosome outside the region targeted by the deletion construct) to identify isolates where pTG1 had integrated into the wild-type copy of the *iglC* gene and not near the previously deleted *iglC* locus. Those isolates were then transformed with pGUTS and again PCR-screened (primers iglC1 and iglC4) to identify clones that yielded one amplicon ~250 bp smaller in size than wild-type LVS. Clones, who had lost the targeted region of *iglC* from both chromosomal loci were cured of pGUTS as aforementioned. The final product was an LVS strain containing two truncated copies of *iglC* (LVS *iglC*-null). Western blot analysis using an anti-IglC antibody (BEI Resources) confirmed loss in expression of the IglC protein in LVS *iglC*-null (data not shown).

The LVS *iglC*-null-complementing construct (pTG28) was generated by PCR amplification of the full-length *iglC* gene, digestion with BamHI and NdeI, and ligation into pGRP that had been previously treated with these same enzymes. This construct was then electroporated into the LVS *iglC*-null mutant.

### Double immunofluorescence microscopy (DIFM)

Differences in intraerythrocytic and extraerythrocytic bacteria were visualized using a double immunofluorescence staining method described previously (Groebel et al., 2009; Horzempa et al., 2011). Human erythrocytes were added to chamber slides (LABTEK) and co-cultured with *F. tularensis* bacteria for 2–3 h. Cells were treated with gentamicin, washed with PBS, and suspended in fixative (2.5% glutaraldehyde or 2% paraformaldehyde plus 0.1% glutaraldehyde in PBS). After washing with PBS again, slides were then blocked with 2.5% bovine serum albumin (BSA; Sigma Aldrich) for 30 min. Subsequently, the cells were probed with polyclonal rabbit anti–*F. tularensis* (BD Biosciences) and PE-conjugated mouse anti-human glycophorin A (CD235a; eBioscience) in 2.5% BSA overnight at 4°C. After multiple washes in PBS with 0.2% Tween 20, the secondary antibodies (Alexa Fluor 350 donkey anti-rabbit IgG and Alexa Fluor 555 donkey anti-mouse IgG; Invitrogen) were added and incubated for at least 1 h at room temperature. The erythrocytes were then washed with 0.2% Tween 20 in PBS and treated with 0.1% Triton X-100 for 10 s to permeabilize. After multiple washes in PBS, the slide was blocked with 2.5% BSA for 30 min at room temperature followed by treatment with rabbit anti-*F. tularensis* for at least 1 h. The cells were washed in 0.2% Tween 20 in PBS and probed with Alexa 488 donkey anti-rabbit for 1 h. Final washes in 0.2% Tween 20 in PBS and PBS alone were performed and then the slide was mounted in ProLong Gold antifade reagent (Invitrogen).

Fluorescence microscopy was carried out using an Olympus IX73 microscope. CellSens Standard software (version 1.7) was used to view the captured images and adjust brightness and contrast uniformly across all images. Adobe Photoshop was used to pseudocolor channels to allow for green-red merge to yellow.

### Statistical analyses

Data were analyzed using statistical tests as indicated in figure legends. GraphPad Prism software was used to determine statistical significance of data generated from this study.

## Results

### Erythrocyte invasion does not contribute to tularemia pathogenesis

Previously published data suggested invasion of erythrocytes by *F. tularensis* may play a role in pathogenesis (Horzempa et al., 2011). To adequately address this possibility, murine erythrocytes were infected with *F. tularensis* Schu S4 and then administered to naïve mice intravenously. As controls, mice were injected with either Schu S4 liberated from erythrocytes or mock-infected erythrocytes mixed with Schu S4 immediately before infection (all three groups were infected with similar levels of bacterial CFU). If erythrocyte invasion enhanced the pathogenesis of *F. tularensis*, then mice infected with bacteria inhabiting RBCs should show accelerated morbidity and mortality. However, all three groups exhibited similar clinical signs of illness and weight loss and succumbed to infection within 7 days (data not shown, *p* = 0.4505). Therefore, erythrocyte invasion is not likely to play a role in the pathogenesis of *F. tularensis*.

### Erythrocyte invasion enhances tick colonization and protects *F. tularensis* from acidic pH associated with tick gut cells

**Figure 1:**
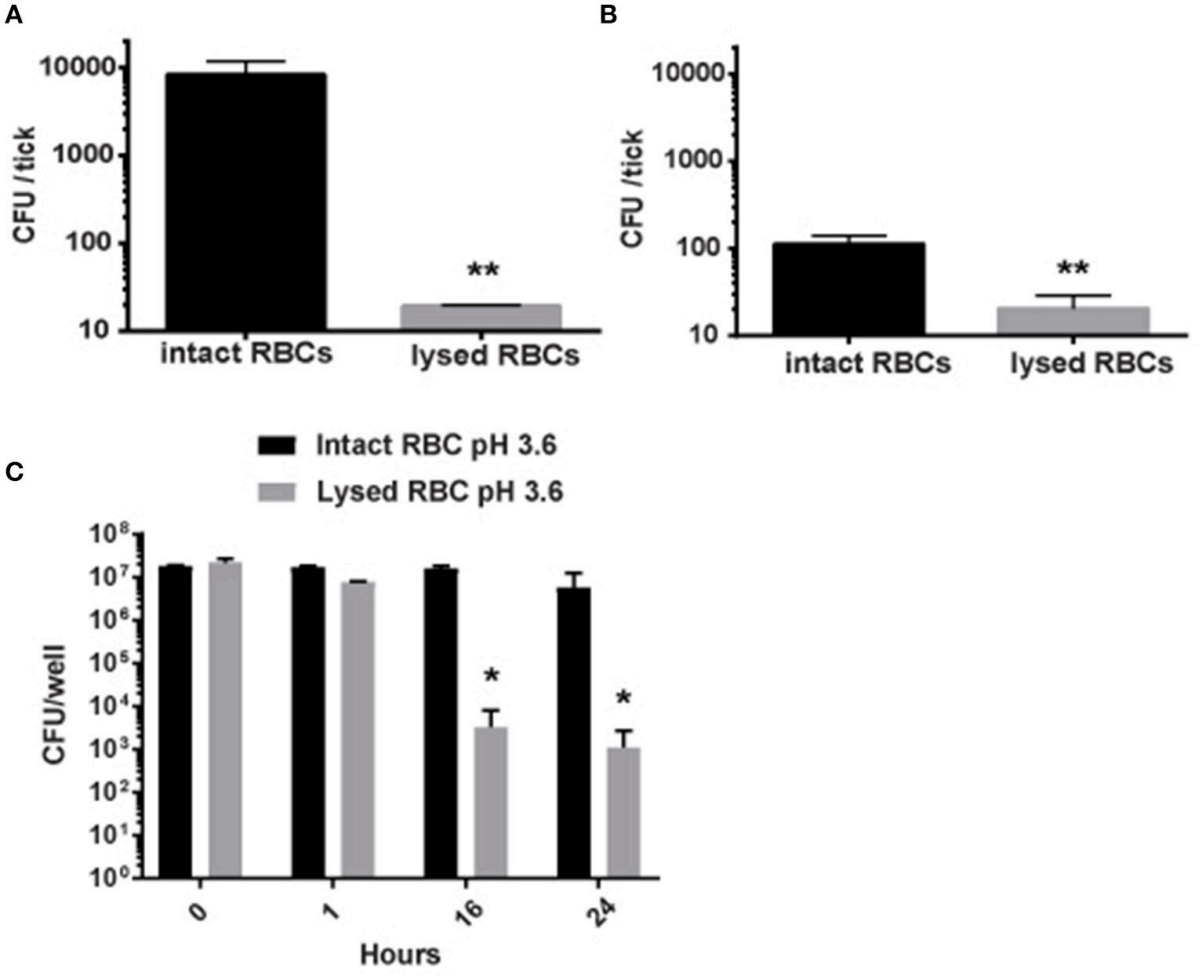
**Residing within erythrocytes enhances the colonization of ticks by ***F. tularensis*** by protecting them from low pH. (A,B)** Erythrocytes incubated with *F. tularensis* LVS bacteria to allow for invasion were either left intact or lysed to liberate intracellular bacteria. Each group was fed to *A. americanum* **(A)** or *I. scapularis* **(B)** ticks using a glass capillary tube. After feeding, the ticks were incubated for 24 h, and then homogenized and plated on media selective for *Francisella*. Increased colonization occurred with intact erythrocytes in comparison to lysed erythrocytes for both *I. scapularis* and *A. americanum* ticks (^**^p < 0.01; unpaired Student's *t*-test). **(C)** *F. tularensis* LVS bacteria were incubated with human erythrocytes for invasion to occur. Erythrocytes were either left intact or lysed to liberate intracellular bacteria. Cells were then incubated in media containing 10% serum at the pH indicated. At designated time points, viable *F. tularensis* bacteria were enumerated and statistically significant differences were determined by two-way ANOVA followed by Sidak's multiple comparisons test (^*^p < 0.05). Data shown are mean ± SD. For all panels, Data are representative of at least three independent experiments in which each individual iteration showed significant differences between the same groups. Each experiment contained at least four separate wells per group.

*Francisella tularensis* is transmitted naturally by blood sucking arthropods—primarily ticks (Reese et al., 2010). Because of the association with erythrocytes, we hypothesized that erythrocyte invasion contributed to tick colonization following a blood meal. In other words, bacteria that resided within RBCs would show an enhanced ability to colonize ticks compared to extracellular bacteria. To test this, we utilized an *in vitro* tick feeding strategy. Ticks (*Amblyomma americanum* or *I. scapularis*) were fed intraerythrocytic *F. tularensis* LVS bacteria, or these same bacteria that had been liberated from RBCs [Lysis of erythrocytes did not reduce bacterial viability (data not shown)]. After feeding and incubation, ticks were homogenized and this material was plated on a medium selective for *F. tularensis*. Ticks fed intact RBCs were more readily colonized than those fed bacteria that had been liberated from erythrocytes (lysed) (Figures 1A,B). The number of viable bacteria was similar in meals containing either intact erythrocytes or liberated bacteria (data not shown). Because inhabiting an erythrocyte enhances colonization of ticks, this suggests that erythrocyte invasion may be a transmission factor. This is based on the premise that an increased bacterial burden in the ticks will increase subsequent transmission to humans.

We next sought to determine why *F. tularensis* LVS bacteria that inhabit erythrocytes are better capable of colonizing ticks. We hypothesized that RBC invasion can protect bacteria against the low pH associated with tick gut cells (Horn et al., 2009). Here, *F. tularensis* LVS bacteria were incubated with human erythrocytes for invasion to occur. RBCs were either left intact or were gently lysed to liberate intracellular bacteria [Lysis of erythrocytes did not reduce bacterial viability (data not shown)]. Cells were then incubated in media containing 10% serum at neutral pH or pH = 3.6 [equivalent to pH of tick gut cells (Horn et al., 2009)]. Bacteria inhabiting erythrocytes or liberated from these host cells survived the entirety of this experiment when incubated in media with a neutral pH (data not shown). Only bacteria inhabiting erythrocytes survived in media adjusted to pH = 3.6 over 24 h (Figure 1C). However, in pH = 3.6 media, the viability of *F. tularensis* LVS bacteria liberated from RBCs dropped significantly at 16 h and thereafter (Figure 1C). These data suggest that erythrocytes provide protection against the acidic pH associated with the digestive cells within the gut of a tick.

To test if erythrocyte invasion provided any protection against the onslaught of proteases associated with blood meal digestion within tick gut cells (Sojka et al., 2013), a similar assay was conducted in which bacteria inhabiting or liberated from erythrocytes were incubated with proteases associated with this process [legumain (0.55 mg/ml), papain (1.2 mg/ml), and cathepsin D (0.1 mg/ml) (Horn et al., 2009)]. However, this protease treatment had no effect on *F. tularensis* LVS viability regardless of the condition of the RBCs (data not shown). Together, these data suggest that invasion of erythrocytes by *F. tularensis* protects the bacterium from the acidic microenvironment of the tick gut allowing for enhanced colonization of this arthropod vector.

### Involvement of type VI secretion system in erythrocyte invasion

**Figure 2:**
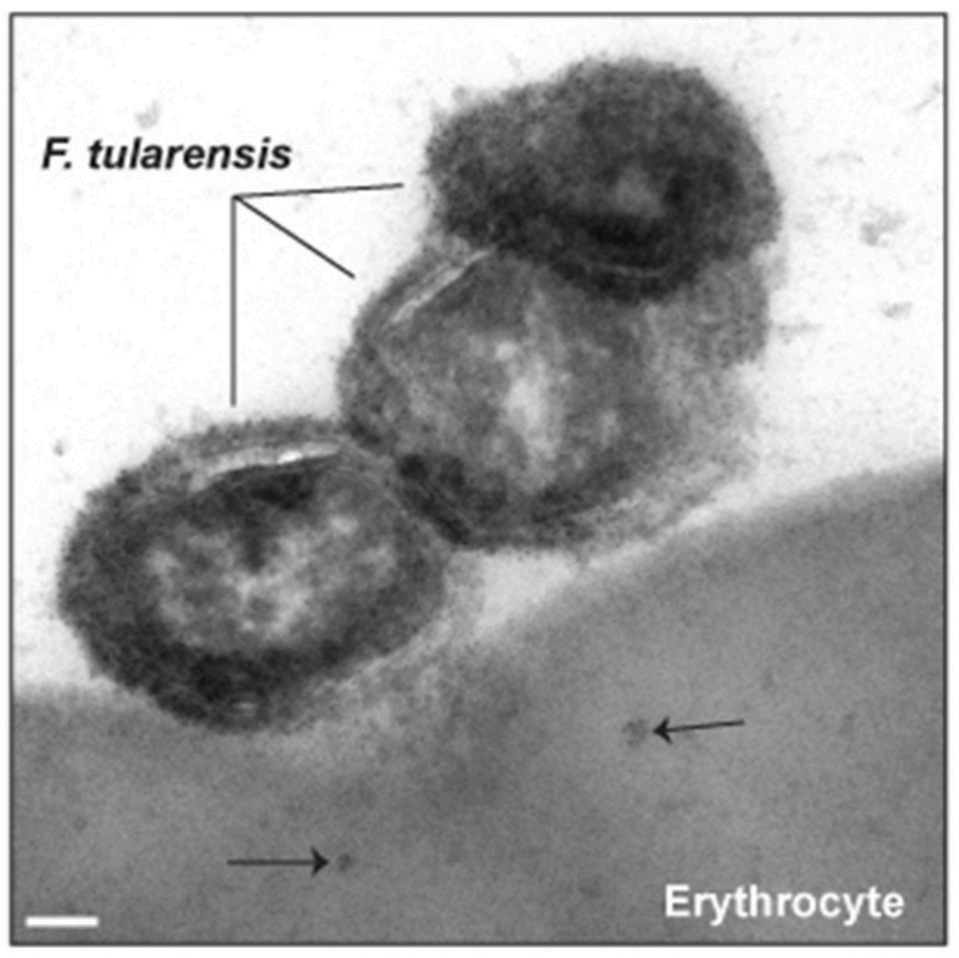
**Bacterial factors are secreted into erythrocytes prior to invasion**. *F. tularensis* Schu S4 was incubated with human erythrocytes for 4 h and processed for immunogold transmission electron microscopy. Ultra-thin sections (70 nm) were probed with anti-*F. tularensis* rabbit antisera and then a 5 nm gold-labeled anti-rabbit secondary antibody. The black arrows designate the location of *F. tularensis* antigen within the erythrocyte. Scale bars represent 100 nm.

Numerous intracellular pathogens utilize protein secretion systems to facilitate invasion of host cells (Eicher and Dehio, 2012; Law et al., 2014; Lee et al., 2014). Therefore, we hypothesized that likewise, erythrocyte invasion by *F. tularensis* was mediated by a protein secretion system. Consistent with this hypothesis, we observed that material derived from *F. tularensis* was found in the cytoplasm of erythrocytes during interaction with these bacteria (Figure 2). As *F. tularensis* possesses type I, II, and VI secretion systems, we conjectured that if secretion was involved in mediating entry into the erythrocytes, then *F. tularensis* genes encoding components of these complexes or regulating their expression would be induced upon culture with erythrocytes. Therefore, gene expression of *F. tularensis* LVS bacteria that had been exposed to RBCs was compared to bacteria incubated in media alone. This RNA-Seq analysis revealed that 14% of *F. tularensis* genes exhibited modest (although statistically significant) changes in their expression in response to erythrocytes (~6% up- and ~8% down-regulated) (see Table S1). Notably, the gene encoding the pathogenicity regulator, macrophage growth locus protein A (MglA) (Baron and Nano, 1998) was upregulated in the presence of RBCs suggesting this induction occurs prior to invasion. Because MglA is required for the expression of the (T6SS) genes (Brotcke et al., 2006), we speculated that the secreted molecules observed in Figure 2 were delivered by this apparatus. If this were true, and if secretion of this material were required for erythrocyte invasion, deletion of *mglA* would abolish this host–pathogen interaction.

**Figure 3:**
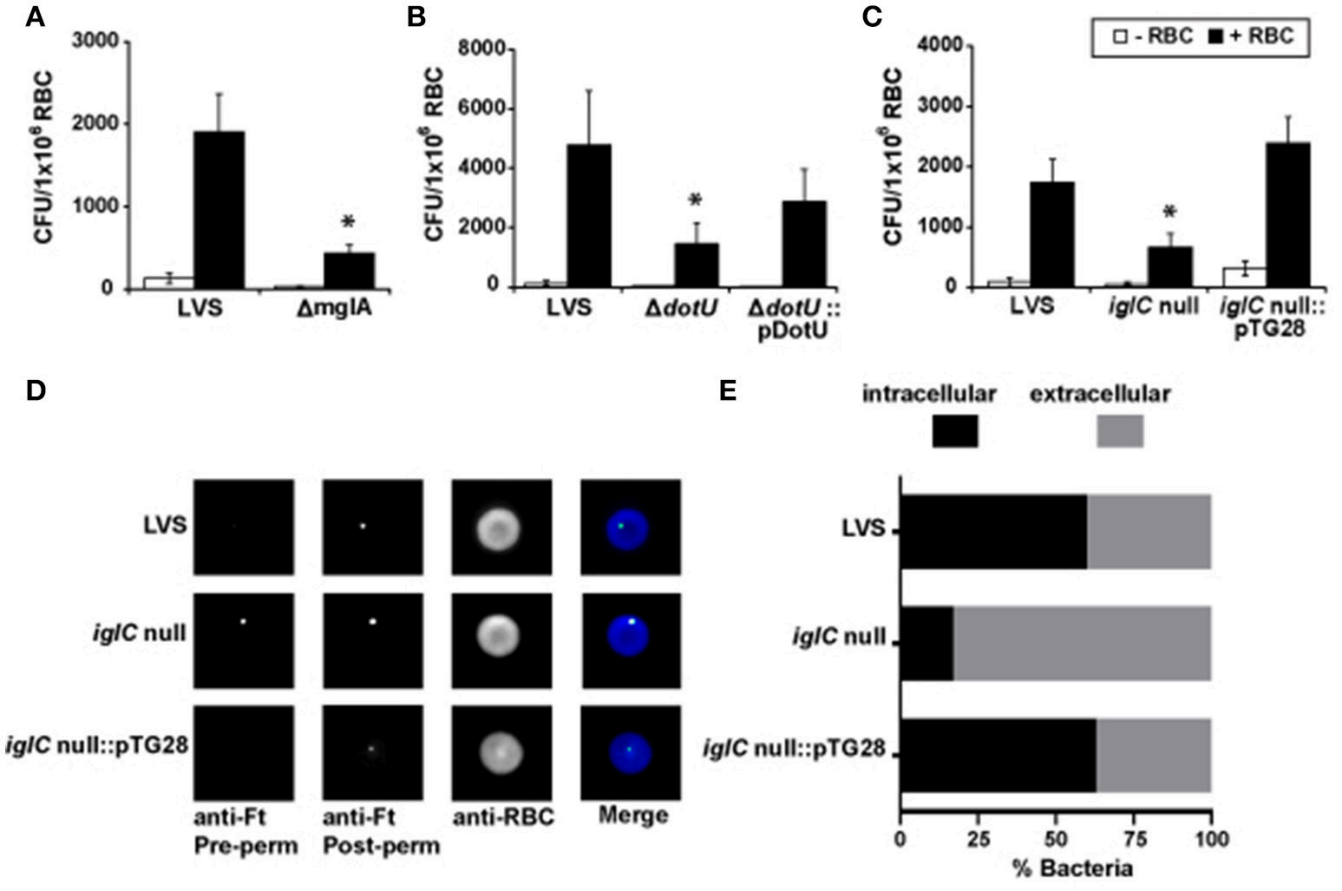
**Erythrocyte invasion by ***F. tularensis*** is mediated by components of the type VI secretion system. (A–C)** Invasion of human erythrocytes by wild-type *F. tularensis* LVS, Δ*mglA*, the type VI secretion system mutants *iglC* null and Δ*dotU*, and their corresponding complemented mutant strains, *iglC* null::pTG28 and Δ*dotU*::pDotU, was measured by a gentamicin protection assay. *F. tularensis* bacteria and erythrocytes (“+RBC”) were co-cultured for 3 h. Cells were then treated with gentamicin, washed, lysed, diluted, and plated to enumerate CFUs (mean ± SEM). Control wells excluded erythrocytes (“−RBC”). Data are representative of five or more independent experiments. For each experiment, we observed significant differences between the same groups. Each iteration contained at least four separate wells per group. ^*^p < 0.05 for LVS vs. Δ*mglA* **(A)**, LVS vs. *iglC* null **(B)**, and LVS vs. Δ*dotU* **(C)**; two-way ANOVA followed by Sidak's multiple comparisons test. **(D,E)** Human erythrocytes incubated with *F. tularensis* LVS, *iglC* null, or *iglC* null::pTG28 were subjected to DIFM. Representative images **(D)** or a quantification of at least five fields of view per group exhibiting bacteria interacting with erythrocytes **(E)** indicate that *iglC* is important for erythrocyte invasion (χ^2^, *p* < 0.0001). Ft, *F. tularensis*; perm, permeabilization; RBC, erythrocyte (red blood cell).

To address this, we conducted a gentamicin protection assay on erythrocytes incubated with a *F. tularensis* LVS *mglA* null mutant (Δ*mglA*). Significantly fewer bacteria were recovered from erythrocytes infected with Δ*mglA* compared to wild-type LVS (Figure 3A) suggesting that MglA-regulated bacterial factors are involved in internalization of the bacterium. As previously mentioned, MglA regulates genes that encode components of the T6SS (Brotcke et al., 2006). To investigate the role of this secretion system in erythrocyte invasion, we evaluated the ability of the two T6SS mutants, Δ*dotU* and *iglC* null, to invade RBCs. DotU is found in the inner membrane of *F. tularensis* and is required for secretion through T6SS (Broms et al., 2012a,b). IglC shares structural similarities to Hcp, the stacking unit of the T6SS inner tube in *Pseudomonas aeruginosa* (de Bruin et al., 2011). Both Δ*dotU* and *iglC* null exhibited reduced invasion of erythrocytes compared to wild-type LVS (Figures 3B,C). To confirm the diminished invasion was due to deletion of *dotU* and *iglC*, RBCs were cultured with trans-complemented strains of LVS Δ*dotU* and *iglC* null and subjected to a gentamicin protection assay. Complementation restored erythrocyte invasion indicating the *F. tularensis* T6SS facilitates erythrocyte invasion (Figures 3B,C).

To further verify the involvement of the T6SS in erythrocyte invasion, we performed DIFM (Groebel et al., 2009) on erythrocytes cultured with wild-type LVS, *iglC* null, or the trans-complemented *iglC* null strain. Similar to the gentamicin protection assay, DIFM distinguishes between intra- and extracellular bacteria (Groebel et al., 2009; Horzempa et al., 2011). The *F. tularensis* LVS *iglC* null mutant bacteria were detected regardless of whether RBCs were permeabilized (Figures 3D,E). However, a significant amount of wild type LVS and the trans-complemented mutant were only observed after permeabilization of the erythrocytes suggesting that these strains successfully invaded red blood cells (Figures 3D,E). These data are consistent with the gentamicin protection assay results observed in Figure 3C and support the conclusion that the T6SS is required for erythrocyte invasion and likely mediates this process.

### Invasion of erythrocytes by *Francisella tularensis* involves an actin-independent process

Host cell cytoskeletal rearrangements are necessary for the uptake of pathogenic bacteria via phagocytosis (Craven et al., 2008; Cossart and Roy, 2010). Further, bacterial invasion of non-phagocytic host cells requires manipulation of cytoskeletal components (Craven et al., 2008). Based on these conventions, we investigated the involvement of erythrocyte cytoskeletal components during invasion by *F. tularensis*.

**Figure 4:**
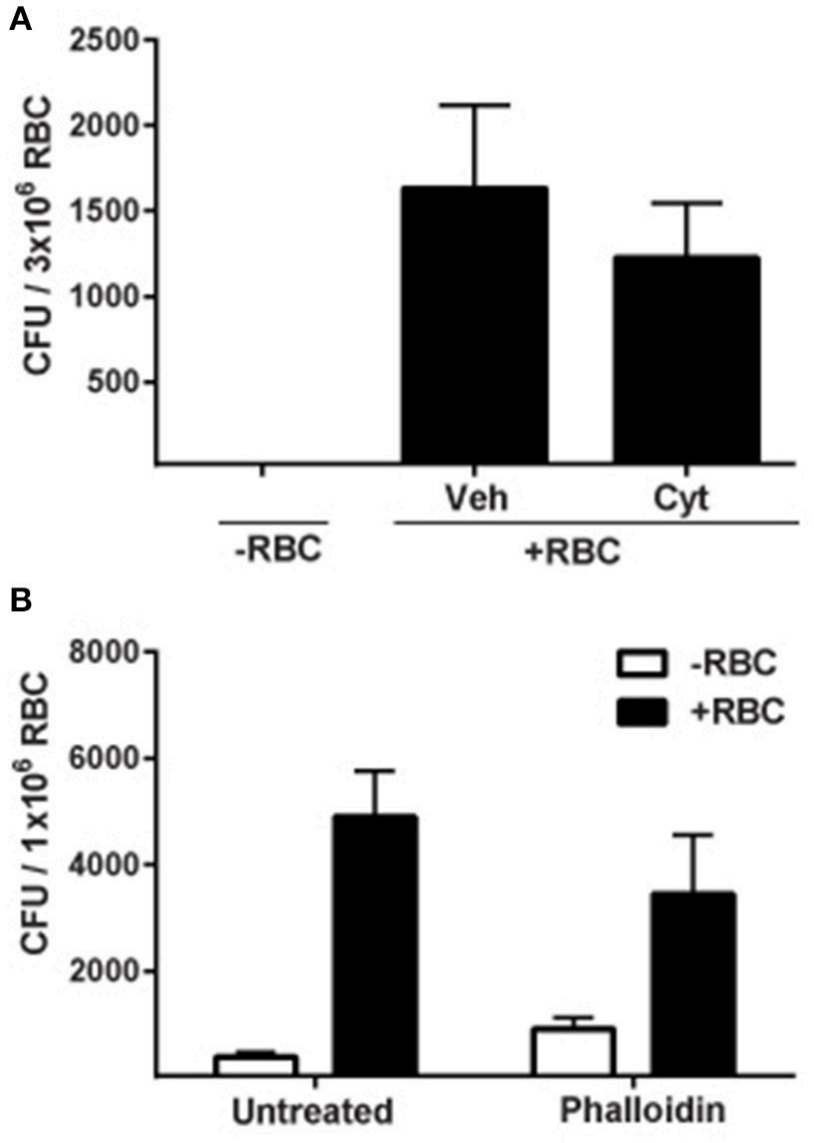
**Actin is not required for invasion of erythrocytes by ***F. tularensis*****. Erythrocytes were treated with 50 μg/mL cytochalasin D (Cyt.) for 30 min **(A)** or 16 μM phalloidin for 20 min **(B)** prior to infection with *F. tularensis* LVS and then subjected to a gentamicin protection assay to measure invasion. Wells lacking erythrocytes served as controls. Data are expressed as mean CFU ± SD for at least three independent experiments. For each experiment, we observed significant differences between the same groups. Each iteration contained at least 4 separate wells per group. RBC, red blood cell; Veh, vehicle, chloroform; Cyt, cytochalasin D.

Both actin polymerization and depolymerization are essential for reorganization of the cytoskeleton (Ribet and Cossart, 2015). To determine whether actin has a role during erythrocyte invasion by *F. tularensis*, erythrocytes were treated with inhibitors of actin polymerization and depolymerization—cytochalasin D (Cyt, 50 μg/mL) and phalloidin (16 μM), respectively. The amount of Cyt used here was 10-times greater than the concentration necessary to inhibit uptake of *F. tularensis* by macrophages (Clemens et al., 2005). A fluorescent analog of phalloidin was used to determine the optimal concentration of the drug that induced visible punctate actin bundles (data not shown). Neither Cyt nor phalloidin treatment inhibited bacterial invasion of human erythrocytes by *F. tularensis* (Figure 4). These data suggest that actin does not likely have a role in erythrocyte invasion by *F. tularensis*.

**Figure 5:**
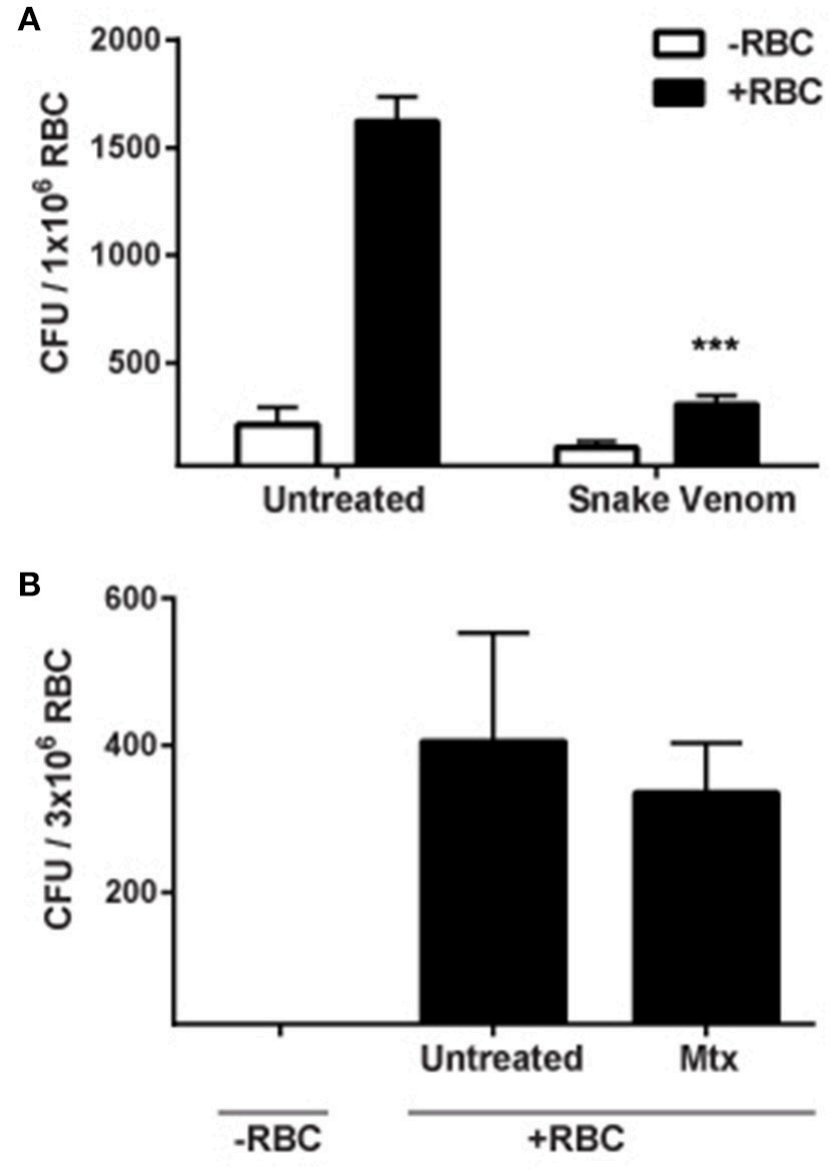
**Spectrin is required for erythrocyte invasion by ***F. tularensis***. (A)** Addition of *P. guttatus* venom reduces invasion of erythrocytes by *F. tularensis* LVS. Erythrocytes were treated with 25 μg/mL *P. guttatus* venom for 20 min prior to infection. Bacteria were co-incubated with the erythrocytes and were subjected to gentamicin protection assay to measure invasion. To confirm that altered membrane fluidics were not responsible for the decreased invasion observed in panel A, erythrocytes were treated with 50 μg/mL mitoxantrone for 30 min prior to infection and subjected to gentamicin protection assay **(B)**. Wells lacking erythrocytes served as controls. For both panels, Data (CFU ± SD) are representative of at least three independent experiments in which each individual iteration showed significant differences between the same groups. Each experiment contained at least 4 separate wells per group. Snake venom (*P. guttatus*); Mtx, mitoxantrone. ^***^p < 0.001; two-way ANOVA followed by Sidak's multiple comparisons test.

Because our results indicated that bacterial entry into erythrocytes is not actin-mediated, we sought to determine the role of spectrin, the major erythrocyte cytoskeletal protein. To address this, RBCs were treated with venom from the Blue-bellied black snake (*P. guttatus*) prior to infection with *F. tularensis*. This venom has been shown to disrupt the cytoskeletal architecture of the erythrocyte by binding Band 3, causing release of spectrin filaments and subsequent aggregation into bundles (Yau et al., 2012). Fewer intracellular *F. tularensis* LVS bacteria were recovered from venom-treated erythrocytes compared to mock-treated controls suggesting that spectrin was required for this process (Figure 5A). This reduced invasion was not due to venom toxicity since the venom did not directly reduce viability of *F. tularensis* bacteria or cause lysis or loss of RBCs over the time allotted for the experiment (data not shown). It is possible that the limited invasion of erythrocytes by *F. tularensis* following venom treatment was due to altered membrane fluidics resulting from disrupted spectrin–phospholipid interactions. To test this hypothesis, erythrocytes were treated with mitoxantrone, a drug that disrupts spectrin–phospholipid interactions (Dubielecka et al., 2006). This treatment did not affect erythrocyte invasion (Figure 5B) suggesting that altered membrane fluidity was not responsible for the reduced level of intracellular bacteria observed in the snake-venom treated RBCs (Figure 5A). This finding is consistent with the interpretation that *F. tularensis* manipulates spectrin to mediate erythrocyte invasion.

## Discussion

Here, we demonstrate that the biological function of erythrocyte invasion is to enhance tick colonization by *F. tularensis*. Residing within an erythrocyte protects the bacterium from the acidic pH of tick gut cells thereby allowing *F. tularensis* to persist within the arthropod.

Previous studies indicated that bacteria utilize hemoglobin to counteract nitrosative stress (Frey et al., 2002). It is therefore possible that hemoglobin from erythrocytes can protect *F. tularensis* bacteria from the oxidative stress associated with the digestive cells of the tick gut. Because *F. tularensis* bacteria can use heme as their sole iron source (Lindgren et al., 2015), future studies should determine whether iron acquired from erythrocytes protect *F. tularensis* bacteria from low pH of the tick gut cells. Alternatively, iron concentration has been shown to be an important regulator of gene expression in *F. tularensis* (Deng et al., 2006). The high content of hemoglobin in the erythrocyte may influence similar changes in *F. tularensis*, allowing the bacterium to adapt to the harsh microenvironment of the tick gut. In this study, we reported the global gene expression changes of *F. tularensis* LVS bacteria in response to human erythrocytes. Some genes previously reported to be regulated by iron concentration were similarly controlled in response to erythrocytes, suggesting that the iron of the red blood cell was the predominant cue. However, as a whole, only a weak inverse correlation (correlation coefficient = −0.3) exists when comparing *F. tularensis* global gene expression changes in response to iron starvation (Deng et al., 2006) to erythrocytes indicating that other RBC cues are serving as transcription regulatory signals. For instance, although MglA transcripts were induced, we also observed that *Francisella* Pathogenicity Island (FPI) genes (a regulon of MglA) were repressed in response to erythrocytes. As the FPI is induced by iron starvation, we hypothesize that heme within erythrocytes triggered repression of these genes, but other red blood cell cues induced expression of MglA (not repressed by iron, Deng et al., 2006). Because data presented here implicated the involvement of the T6SS (products of the FPI) in erythrocyte invasion, MglA induction by erythrocyte cues was likely sufficient to overcome the complete repression of FPI genes in response to abundant iron. In support of this interpretation, three other MglA-activated genes (2-isopropylmalate synthase, β-lactamase, and D-alanyl-D-alanine carboxypeptidase (Penicillin binding protein) family protein) were induced in the presence of RBCs. Therefore, in addition to providing a list of candidate genes that may be involved in erythrocyte invasion, the RNA profiling study presented here also contributes to our understanding of *F. tularensis* virulence factors and the MglA regulon.

Entry of *F. tularensis* into erythrocytes is dependent upon the (T6SS). The specific identity of the secreted proteins required for invasion has not been determined, however, it is likely one or several effectors of the *Francisella* T6SS are involved. One study measuring the translocation of TEM-1 reporter fusions by *F. tularensis* LVS identified several proteins encoded within the FPI as T6SS substrates, including IglE, IglF, IglI, IglJ, and PdpE (Broms et al., 2012b). A more recent study identified two additional FPI-encoded proteins as T6SS secreted effectors, PdpC and PdpD, along with two proteins encoded outside the FPI, OpiA, and OpiB (Eshraghi et al., 2016). IglE, IglF, IglI, IglJ, and PdpE were not identified as T6SS substrates by the proteome-wide approach to find T6SS effectors by Eshraghi et al. which could mean that technical aspects regarding this methodology precluded the identification of these proteins. On the other hand, the utilization of reporter constructs to identify secreted effectors of the T6SS by Broms et al. could have led to the discovery of false positives. Future work will test the contribution of potential effectors along with others in facilitating invasion of erythrocytes by *F. tularensis*, particularly their role in modifying spectrin.

Alternatively, other secretion systems may be involved in mediating uptake of *F. tularensis* by erythrocytes. Both Type I and II secretion systems are produced by *F. tularensis* (Rowe and Huntley, 2015). Mutational analysis of genes encoding components of the type II secretion system (T2SS) revealed that this apparatus is not involved in erythrocyte invasion (data not shown). Preliminary experiments with the T1SS mutant Δ*hlyD* (FTL_0687) show a defect in erythrocyte invasion compared to wild-type LVS suggesting a role for the T1SS (data not shown). Interestingly, in other bacteria, hemolysins are secreted through the T1SS (45). Since *F. tularensis* LVS is not hemolytic, direct involvement of the T1SS could indicate an evolutionary step in which the *Francisella* T1SS effectors shifted from mediating hemolysis to intracellular residence.

Cytoskeletal rearrangement is also essential for internalization of *F. tularensis* into erythrocytes, similar to macrophages and non-phagocytic cells. However, treatment of erythrocytes with inhibitors of actin polymerization and depolymerization failed to block invasion by *F. tularensis*, thus, highlighting a unique actin-independent mechanism of bacterial entry. Subsequent experiments with snake venom from *P. guttatus* identified spectrin as the primary cytoskeletal protein responsible for mediating uptake of *F. tularensis* by erythrocytes. As *P. guttatus* venom interacts directly with Band 3 to disrupt the spectrin network (Yau et al., 2012), it is possible that *F. tularensis* bacteria either utilize Band 3 to manipulate spectrin or secrete effectors that bind spectrin to mediate entry. Additionally, a proteomic analysis of erythrocytes revealed the presence of trace amounts of clathrin (Goodman et al., 2007), a key protein involved in the formation of endocytic vesicles. Receptor-mediated endocytosis has not been observed in mature human RBCs (Schekman and Singer, 1976). However, *F. tularensis* may secrete effectors capable of manipulating clathrin to induce an endocytic event allowing for bacterial invasion.

## Ethics statement

This study was carried out in accordance with the recommendations of the University of Pittsburgh's Institutional Animal Care and Use Committee (IACUC). The protocol was approved by the University of Pittsburgh IACUC.

## Author contributions

DMS, RB, TR, AH, and JH wrote the manuscript. DMS, RB, TR, AH, LM, MF, TG, JB, DR, JMF, DBS, JD, JF, SR, DAP, and JH conducted the experiments. DMS, TG, and AS constructed the plasmids and mutant strains, DMS, RB, TR, AH, LM, MF, TG, JB, JD, JF, DBS, SR, DAP, and JH analyzed the data. DMS, RB, TR, AH, AS, DR, JD, DAP, and JH edited the manuscript. DMS and JH envisioned and designed the study. JH oversaw the work. All authors read and approved the final version of this manuscript.

## Funding

This work was funded by a grant through the NASA WV Space Grant Consortium (NNX10AK62H), an Institutional Development Award (IDeA) from the National Institute of General Medical Sciences of the National Institutes of Health (P20GM103434) which funds WV-INBRE program and the Marshall University Genomics Core Facility, a grant from the National Institutes of Health, National Institute of Allergy and Infectious Diseases (5K22AI087703), and funding from the WV Research Challenge Fund (HEPC.dsr.14.13).

### Conflict of interest statement

The authors declare that the research was conducted in the absence of any commercial or financial relationships that could be construed as a potential conflict of interest.

## References

[B1] Barker J. R. Klose K. E. (2007). Molecular and genetic basis of pathogenesis in *Francisella tularensis*. Ann. N. Y. Acad. Sci. 1105, 138–159. 10.1196/annals.1409.010 17395737

[B2] Baron G. S. Nano F. E. (1998). MglA and MglB are required for the intramacrophage growth of *Francisella novicida*. Mol. Microbiol. 29, 247–259. 10.1046/j.1365-2958.1998.00926.x 9701818

[B3] Bosio C. M. Dow S. W. (2005). *Francisella tularensis* induces aberrant activation of pulmonary dendritic cells. J. Immunol. 175, 6792–6801. 10.4049/jimmunol.175.10.6792 16272336

[B4] Boyum A. (1964). Separation of white blood cells. Nature 204, 793–794. 10.1038/204793a0 14235685

[B5] Boyum A. (1968). Separation of leukocytes from blood and bone marrow. Introduction. Scand. J. Clin. Lab. Invest. Suppl. 97:7. 5707208

[B6] Broms J. E. Meyer L. Lavander M. Larsson P. Sjostedt A. (2012a). DotU and VgrG, core components of type VI secretion systems, are essential for Francisella LVS pathogenicity. PLoS ONE 7:e34639. 10.1371/journal.pone.0034639 22514651 PMC3326028

[B7] Broms J. E. Meyer L. Sun K. Lavander M. Sjostedt A. (2012b). Unique substrates secreted by the type VI secretion system of *Francisella tularensis* during intramacrophage infection. PLoS ONE 7:e50473. 10.1371/journal.pone.0050473 23185631 PMC3502320

[B8] Brotcke A. Weiss D. S. Kim C. C. Chain P. Malfatti S. Garcia E.. (2006). Identification of MglA-regulated genes reveals novel virulence factors in *Francisella tularensis*. Infect. Immun. 74, 6642–6655. 10.1128/IAI.01250-06 17000729 PMC1698089

[B9] Brown H. E. Yates K. F. Dietrich G. MacMillan K. Graham C. B. Reese S. M.. (2011). An acarologic survey and *Amblyomma americanum* distribution map with implications for tularemia risk in Missouri. Am. J. Trop. Med. Hyg. 84, 411–419. 10.4269/ajtmh.2011.10-0593 21363979 PMC3042817

[B10] Carter R. (2001). Transmission blocking malaria vaccines. Vaccine 19, 2309–2314. 10.1016/S0264-410X(00)00521-1 11257353

[B11] Castellaw A. H. Showers J. Goddard J. Chenney E. F. Varela-Stokes A. S. (2010). SDetection of vector-borne agents in lone star ticks, *Amblyomma americanum* (Acari: Ixodidae), from Mississippi. J. Med. Entomol. 47, 473–476. 10.1093/jmedent/47.3.473 20496596

[B12] Chakrabarti A. Kelkar D. A. Chattopadhyay A. (2006). Spectrin organization and dynamics: new insights. Biosci. Rep. 26, 369–386. 10.1007/s10540-006-9024-x 17029004

[B13] Chamberlain R. E. (1965). Evaluation of live tularemia vaccine prepared in a chemically defined medium. Appl. Microbiol. 13, 232–235. 14325885 10.1128/am.13.2.232-235.1965 PMC1058227

[B14] Clemens D. L. Lee B. Y. Horwitz M. A. (2005). *Francisella tularensis* enters macrophages via a novel process involving pseudopod loops. Infect. Immun. 73, 5892–5902. 10.1128/IAI.73.9.5892-5902.2005 16113308 PMC1231130

[B15] Cossart P. Roy C. R. (2010). Manipulation of host membrane machinery by bacterial pathogens. Curr. Opin. Cell Biol. 22, 547–554. 10.1016/j.ceb.2010.05.006 20542678 PMC2975266

[B16] Craven R. R. Hall J. D. Fuller J. R. Taft-Benz S. Kawula T. H. (2008). *Francisella tularensis* invasion of lung epithelial cells. Infect. Immun. 76, 2833–2842. 10.1128/IAI.00043-08 18426871 PMC2446690

[B17] de Bruin O. M. Duplantis B. N. Ludu J. S. Hare R. F. Nix E. B. Schmerk C. L.. (2011). The biochemical properties of the Francisella pathogenicity island (FPI)-encoded proteins IglA, IglB, IglC, PdpB and DotU suggest roles in type VI secretion. Microbiology 157(Pt 12), 3483–3491. 10.1099/mic.0.052308-0 21980115 PMC3352279

[B18] Dehio C. (2008). Infection-associated type IV secretion systems of Bartonella and their diverse roles in host cell interaction. Cell. Microbiol. 10, 1591–1598. 10.1111/j.1462-5822.2008.01171.x 18489724 PMC2610397

[B19] Deng K. Blick R. J. Liu W. Hansen E. J. (2006). Identification of *Francisella tularensis* genes affected by iron limitation. Infect. Immun. 74, 4224–4236. 10.1128/IAI.01975-05 16790797 PMC1489736

[B20] Dennis D. T. Inglesby T. V. Henderson D. A. Bartlett J. G. Ascher M. S. Eitzen E.. (2001). Tularemia as a biological weapon: medical and public health management. JAMA 285, 2763–2773. 10.1001/jama.285.21.2763 11386933

[B21] Dhermy D. Schrevel J. Lecomte M. C. (2007). Spectrin-based skeleton in red blood cells and malaria. Curr. Opin. Hematol. 14, 198–202. 10.1097/MOH.0b013e3280d21afd 17414207

[B22] Dobler G. Fingerle V. Hagedorn P. Pfeffer M. Silaghi C. Tomaso H.. (2014). [Threat of transmission of infectious pathogens by Ixodes ricinus ticks in Germany]. Bundesgesundheitsblatt Gesundheitsforschung Gesundheitsschutz 57, 541–548. 10.1007/s00103-013-1921-0 24781911

[B23] Dubielecka P. M. Trusz A. Diakowski W. Grzybek M. Chorzalska A. Jazwiec B.. (2006). Mitoxantrone changes spectrin-aminophospholipid interactions. Mol. Membr. Biol. 23, 235–243. 10.1080/09687860600601643 16785207

[B24] Eicher S. C. Dehio C. (2012). Bartonella entry mechanisms into mammalian host cells. Cell. Microbiol. 14, 1166–1173. 10.1111/j.1462-5822.2012.01806.x 22519749

[B25] Ellis J. Oyston P. C. Green M. Titball R. W. (2002). Tularemia. Clin. Microbiol. Rev. 15, 631–646. 10.1128/CMR.15.4.631-646.2002 12364373 PMC126859

[B26] Eshraghi A. Kim J. Walls A. C. Ledvina H. E. Miller C. N. Ramsey K. M.. (2016). Secreted effectors encoded within and outside of the Francisella pathogenicity island promote intramacrophage growth. Cell Host Microbe 20, 573–583. 10.1016/j.chom.2016.10.008 27832588 PMC5384264

[B27] Feldman K. A. Enscore R. E. Lathrop S. L. Matyas B. T. McGuill M. Schriefer M. E. (2001). An outbreak of primary pneumonic tularemia on Martha's Vineyard. N. Engl. J. Med. 345, 1601–1606. 10.1056/NEJMoa011374 11757506

[B28] Ferrante A. Thong Y. H. (1980). Optimal conditions for simultaneous purification of mononuclear and polymorphonuclear leucocytes from human blood by the Hypaque-Ficoll method. J. Immunol. Methods 36, 109–117. 10.1016/0022-1759(80)90036-8 7430646

[B29] Frey A. D. Farres J. Bollinger C. J. Kallio P. T. (2002). Bacterial hemoglobins and flavohemoglobins for alleviation of nitrosative stress in *Escherichia coli*. Appl. Environ. Microbiol. 68, 4835–4840. 10.1128/AEM.68.10.4835-4840.2002 12324328 PMC126413

[B30] Fujita H. Watanabe Y. Sato T. Ohara Y. Homma M. (1993). The entry and intracellular multiplication of *Francisella tularensis* in cultured cells: its correlation with virulence in experimental mice. Microbiol. Immunol. 37, 837–842. 10.1111/j.1348-0421.1993.tb01713.x 8295562

[B31] Goodman S. R. Kurdia A. Ammann L. Kakhniashvili D. Daescu O. (2007). The human red blood cell proteome and interactome. Exp. Biol. Med. (Maywood). 232, 1391–1408. 10.3181/0706-MR-156 18040063

[B32] Groebel K. Hoelzle K. Wittenbrink M. M. Ziegler U. Hoelzle L. E. (2009). Mycoplasma suis invades porcine erythrocytes. Infect. Immun. 77, 576–584. 10.1128/IAI.00773-08 19015255 PMC2632055

[B33] Gurcan S. (2014). Epidemiology of tularemia. Balkan Med. J. 31, 3–10. 10.5152/balkanmedj.2014.13117 25207161 PMC4115998

[B34] Hall J. D. Craven R. R. Fuller J. R. Pickles R. J. Kawula T. H. (2007). *Francisella tularensis* replicates within alveolar type II epithelial cells *in vitro* and *in vivo* following inhalation. Infect. Immun. 75, 1034–1039. 10.1128/IAI.01254-06 17088343 PMC1828526

[B35] Hall J. D. Woolard M. D. Gunn B. M. Craven R. R. Taft-Benz S. Frelinger J. A.. (2008). Infected-host-cell repertoire and cellular response in the lung following inhalation of *Francisella tularensis* Schu S4, LVS, or U112. Infect. Immun. 76, 5843–5852. 10.1128/IAI.01176-08 18852251 PMC2583552

[B36] Hazlett K. R. Caldon S. D. McArthur D. G. Cirillo K. A. Kirimanjeswara G. S. Magguilli M. L.. (2008). Adaptation of *Francisella tularensis* to the mammalian environment is governed by cues which can be mimicked *in vitro*. Infect. Immun. 76, 4479–4488. 10.1128/IAI.00610-08 18644878 PMC2546835

[B37] Horn M. Nussbaumerova M. Sanda M. Kovarova Z. Srba J. Franta Z.. (2009). Hemoglobin digestion in blood-feeding ticks: mapping a multipeptidase pathway by functional proteomics. Chem. Biol. 16, 1053–1063. 10.1016/j.chembiol.2009.09.009 19875079 PMC2801564

[B38] Horzempa J. Carlson P. E. Jr. O'Dee D. M. Shanks R. M. Nau G. J. (2008). Global transcriptional response to mammalian temperature provides new insight into *Francisella tularensis* pathogenesis. BMC Microbiol. 8:172. 10.1186/1471-2180-8-172 18842136 PMC2576331

[B39] Horzempa J. O'Dee D. M. Shanks R. M. Nau G. J. (2010a). *Francisella tularensis* DeltapyrF mutants show that replication in non-macrophages is sufficient for pathogenesis *in vivo*. Infect. Immun. 78, 2607–2619. 10.1128/IAI.00134-10 20385757 PMC2876533

[B40] Horzempa J. O'Dee D. M. Stolz D. B. Franks J. M. Clay D. Nau G. J. (2011). Invasion of erythrocytes by *Francisella tularensis*. J. Infect. Dis. 204, 51–59. 10.1093/infdis/jir221 21628658 PMC3105038

[B41] Horzempa J. Shanks R. M. Brown M. J. Russo B. C. O'Dee D. M. Nau G. J. (2010b). Utilization of an unstable plasmid and the I-SceI endonuclease to generate routine markerless deletion mutants in *Francisella tularensis*. J. Microbiol. Methods 80, 106–108. 10.1016/j.mimet.2009.10.013 19879904 PMC3034693

[B42] Kocan K. M. de la Fuente J. Blouin E. F. (2007). Targeting the tick/pathogen interface for developing new anaplasmosis vaccine strategies. Vet. Res. Commun. 31(Suppl. 1), 91–96. 10.1007/s11259-007-0070-z 17682853

[B43] Langmead B. Trapnell C. Pop M. Salzberg S. L. (2009). Ultrafast and memory-efficient alignment of short DNA sequences to the human genome. Genome Biol. 10:R25. 10.1186/gb-2009-10-3-r25 19261174 PMC2690996

[B44] Law H. T. Sriram A. Fevang C. Nix E. B. Nano F. E. Guttman J. A. (2014). IglC and PdpA are important for promoting Francisella invasion and intracellular growth in epithelial cells. PLoS ONE 9:e104881. 10.1371/journal.pone.0104881 25115488 PMC4130613

[B45] Lee J. H. Park H. Park Y. H. (2014). Molecular mechanisms of host cytoskeletal rearrangements by *Shigella invasins*. Int. J. Mol. Sci. 15, 18253–18266. 10.3390/ijms151018253 25310650 PMC4227214

[B46] Li H. Handsaker B. Wysoker A. Fennell T. Ruan J. Homer N.. (2009). The sequence alignment/map format and SAMtools. Bioinformatics 25, 2078–2079. 10.1093/bioinformatics/btp352 19505943 PMC2723002

[B47] Lindgren H. Lindgren L. Golovliov I. Sjostedt A. (2015). Mechanisms of heme utilization by *Francisella tularensis*. PLoS ONE 10:e0119143. 10.1371/journal.pone.0119143 25756756 PMC4355490

[B48] Love M. I. Huber W. Anders S. (2014). Moderated estimation of fold change and dispersion for RNA-seq data with DESeq2. Genome Biol. 15, 550. 10.1186/s13059-014-0550-8 25516281 PMC4302049

[B49] Morner T. (1992). The ecology of tularaemia. Rev. Sci. Tech. 11, 1123–1130. 10.20506/rst.11.4.657 1305858

[B50] Nigrovic L. E. Wingerter S. L. (2008). Tularemia. Infect Dis. Clin. North Am. 22, 489–504, ix. 10.1016/j.idc.2008.03.004 18755386

[B51] Noble P. B. Cutts J. H. (1967). Separation of blood leukocytes by Ficoll gradient. Can. Vet. J. 8, 110–111. 6046425 PMC1696854

[B52] Palek J. Liu S. C. (1980). Red cell membrane skeleton: structure-function relationships. Prog. Clin. Biol. Res. 43, 21–44. 6999502

[B53] Petersen J. M. Mead P. S. Schriefer M. E. (2009). *Francisella tularensis*: an arthropod-borne pathogen. Vet. Res. 40:7. 10.1051/vetres:2008045 18950590 PMC2695023

[B54] Qin A. Mann B. J. (2006). Identification of transposon insertion mutants of *Francisella tularensis* tularensis strain Schu S4 deficient in intracellular replication in the hepatic cell line HepG2. BMC Microbiol. 6:69. 10.1186/1471-2180-6-69 16879747 PMC1557513

[B55] Rechav Y. Zyzak M. Fielden L. J. Childs J. E. (1999). Comparison of methods for introducing and producing artificial infection of ixodid ticks (Acari: Ixodidae) with *Ehrlichia chaffeensis*. J. Med. Entomol. 36, 414–419. 10.1093/jmedent/36.4.414 10467766

[B56] Reese S. M. Dietrich G. Dolan M. C. Sheldon S. W. Piesman J. Petersen J. M.. (2010). Transmission dynamics of *Francisella tularensis* subspecies and clades by nymphal *Dermacentor variabilis* (Acari: Ixodidae). Am. J. Trop. Med. Hyg. 83, 645–652. 10.4269/ajtmh.2010.10-0127 20810833 PMC2929064

[B57] Ribet D. Cossart P. (2015). How bacterial pathogens colonize their hosts and invade deeper tissues. Microbes Infect. 17, 173–183. 10.1016/j.micinf.2015.01.004 25637951

[B58] Rowe H. M. Huntley J. F. (2015). From the outside-in: the *Francisella tularensis* envelope and virulence. Front. Cell. Infect. Microbiol. 5:94. 10.3389/fcimb.2015.00094 26779445 PMC4688374

[B59] Schekman R. Singer S. J. (1976). Clustering and endocytosis of membrane receptors can be induced in mature erythrocytes of neonatal but not adult humans. Proc. Natl. Acad. Sci. U.S.A. 73, 4075–4079. 10.1073/pnas.73.11.4075 1069294 PMC431333

[B60] Schulein R. Seubert A. Gille C. Lanz C. Hansmann Y. Piemont Y.. (2001). Invasion and persistent intracellular colonization of erythrocytes. A unique parasitic strategy of the emerging pathogen Bartonella. J. Exp. Med. 193, 1077–1086. 10.1084/jem.193.9.1077 11342592 PMC2193435

[B61] Shaw M. K. (2003). Cell invasion by *Theileria sporozoites*. Trends Parasitol. 19, 2–6. 10.1016/S1471-4922(02)00015-6 12488213

[B62] Sjostedt A. (2007). Tularemia: history, epidemiology, pathogen physiology, and clinical manifestations. Ann. N. Y. Acad. Sci. 1105, 1–29. 10.1196/annals.1409.009 17395726

[B63] Sojka D. Franta Z. Horn M. Caffrey C. R. Mares M. Kopacek P. (2013). New insights into the machinery of blood digestion by ticks. Trends Parasitol. 29, 276–285. 10.1016/j.pt.2013.04.002 23664173

[B64] Yau T. W. Kuchel R. P. Koh J. M. Szekely D. Mirtschin P. J. Kuchel P. W. (2012). Cytoskeletal rearrangements in human red blood cells induced by snake venoms: light microscopy of shapes and NMR studies of membrane function. Cell Biol. Int. 36, 87–97. 10.1042/CBI20110012 21933154

